# A Possible Next Covid-19 Pandemic: The Violence Against Women and Its Psychiatric Consequences

**DOI:** 10.3389/fpsyt.2021.650671

**Published:** 2021-06-25

**Authors:** Domenico De Berardis, Giulia Gianfelice, Michele Fornaro, Federica Vellante, Antonio Ventriglio, Gabriella Marini, Mauro Pettorruso, Giovanni Martinotti, Silvia Fraticelli, Massimo Di Giannantonio

**Affiliations:** ^1^National Health Service, Department of Mental Health, Psychiatric Service for Diagnosis and Treatment, Hospital “G. Mazzini, ” Azienda Sanitaria Locale 4, Teramo, Italy; ^2^Department of Neurosciences and Imaging, Chair of Psychiatry, University “G. D'Annunzio” Chieti, Chieti, Italy; ^3^Department of Psychiatry, Federico II University, Naples, Italy; ^4^Department of Psychiatry, University of Foggia, Foggia, Italy; ^5^ANAAO Assomed Syndicate, Women Section, Hospice and Palliative Therapy Unit, ASL Teramo, Teramo, Italy

**Keywords:** COVID-19, violence, women, maltreatment, psychiatric disorders

## Introduction

Covid-19 has left the aftermath characterized by an increase of psychological distress due to several causes, such as social distancing, fear of contagion, less utilization of healthcare resources, and last but not least, the lockdown in several countries. The lockdown has negatively affected the psychological well-being and has favored the emergence or re-exacerbation of psychiatric disorders (1).

The “forced” lockdown has obliged families to live together under the same home and, often, in a restricted objective and personal spaces. The problem now is that, due to the lockdown measures, families are forced to live together 24/7 compared to the time they would spend all together before the pandemic. This has increased the possibility of conflicts, quarrels, and episodes of interpersonal violence. Moreover, one of the most critical issues of the lockdown has involved the cohabitation of families that were problematic and, particularly, couples with marriage problems and were also approaching a divorce or a separation before COVID-19 (2). Sadly, many females may have paid the higher price of this forced cohabitation in a global context that was, even before, highly alarming regarding the violence against women (VAW) and girls.

Moreover, the VAW was still a worldwide and community health problem even before the COVID-19 pandemic, but it was often neglected in this particular period (as it happened for mental health as well), and therefore our opinion paper aims to draw attention to it.

## The Violence Against the Women as a Worldwide Problem During COVID-19 Pandemic

The Declaration on the Elimination of Violence Against Women, adopted by the United Nations General Assembly in 1993, defined VAW as “any act of gender-based violence that results in, or is likely to result in, physical, sexual, or psychological harm or suffering to women, including threats of such acts, coercion or arbitrary deprivation of liberty, whether occurring in public or private life.” (3) Hence, VAW is expressed through physical, sexual, emotional, and economic methods. The universal categories of VAW are domestic and sexual violence, sexual harassment, and psychological forms of abuse (4).

Available shreds of evidence showed that almost one in three women has suffered physical and/or sexual violence from a close partner in her life (5). In 2018, the WHO conducted an analysis of prevalence analyzing data from 2000 to 2018 in 161 countries and found that worldwide, nearly 1 in 3, or 30%, of women have been subjected to physical and/or sexual violence by an intimate partner or non-partner. In addition, women may face greater vulnerability to multiple forms of discrimination (older women, those living with disabilities, LGBTQI and trans women, migrants, displaced and refugee women, victims of armed conflict, indigenous women, etc.) (6). Finally, there is evidence that the violence against women might increase after natural disasters (7). For example, an increase in psychological violence and sexual harassment against women was reported within the communities of several Iranian regions struck by earthquakes and floods between 2012 and 2013 (8). As well, another study conducted in Japan has yielded similar results (9).

However, this phenomenon has become a cause of concern during and after the COVID-19 lockdown in many countries worldwide (10).

Aguero (11) reported an increase in calls to the helpline for violence against women in Peru after stay-at-home policies in mid-March, with a 48 percent rise since the pandemic and still increasing over time. In the Hubei province of China, the police reports have pointed out the triplicating of domestic violence events against women in February 2020 than February 2019, guessing that 90% were related to the Covid-19 and lockdown (12). In the United Kingdom, a pioneering project against women violence reported 16 deaths between March 23 and April 12, 2020, which was almost doubled compared with the mean rate in the preceding 10 years (12). Finally, Jetelina et al. (13) conducted a cross-sectional study to evaluate intimate partner violence (IPV) severity and the categories of victimization during the initial stages of the COVID-19 pandemic in the USA. They found that sexual and physical violence increased during the initial stages of the pandemic, but sexual violence considerably worsened among victims as a potential effect of spending more hours of the day at home.

Speaking at a press briefing on May 7, 2020, Hans Kluge, regional director of WHO Europe, reported that “WHO is deeply troubled by the reports from many countries, including Belgium, Bulgaria, France, Ireland, Russian Federation, Spain, UK, and others of increases in interpersonal violence, including violence against women and men, by an intimate partner and against children—because of the COVID-19 response” (14).

In Italy, it has been reported by the Italian network of shelters for women subjected to gender-based violence (Donne in rete contro la violenza, D.i.Re) that 2,956 women asked for help from anti-violence centers, with 979 (33.1%) asking help for the first time, between April 6 and May 3, 2020 (15). However, before these dates, the number was lower, and a possible explanation may be that the abused women were under the control of the perpetrators, unable to ask for help and that several anti-violence centers were closed due to the pandemic.

Moreover, it is worthy to note that, during the lockdown, has been reported increased use of alcohol and other substances, leading, in some cases, to Alcohol or Substance Use Disorders, even in subjects without these problems before lockdown (16). This increase in alcohol and substance use or abuse may have triggered the violence in perpetrators or exacerbated previous marriage problems. Moreover, job loss, monetary problems, foodstuff uncertainty, and privation of social support may have contributed to increased violence odds by men against women (17). Moreover, a recent study pointed out that the lockdown due to the COVID-19 spread could foster a dysregulation of biological and social rhythms and, consequently, the occurrence of Bipolar Disorders, and this might be a cause of increased alcohol and other substances use and abuse (18).

What can we expect during Phases Two (i.e., the loosening of lockdown measures)? First, there are some observations that a constant increase in violence reporting against the women happened during the lockdown (19). This may overwhelm the anti-violence center leading to potential difficulty addressing this phenomenon and supporting the victims. Thus, many women may not receive help and be left to cope with these terrible situations independently.

## Psychiatric Consequences of Violence Against Women During COVID-19 Pandemic

It has been demonstrated that the psychiatric consequences of violence against the women (when the victim survives, as femicide is frequent) are the development of Adjustment Disorders, Acute Stress Disorder, and Post-Traumatic Stress Disorder that may also complicate with other several conditions as Major Depression, substance abuse and suicidal behaviors (20), even in women without a prior history of psychiatric disorders.

Besides, VAW and IPV might increase in pregnancy/postpartum or has deleterious effects on mother-infant bonding and child outcomes (21). In a recent systematic review, Pastor-Moreno et al. (22) showed a relation between psychological IPV and adverse outcomes, including premature rupture of membranes, preterm birth, urinary tract infections, and late entry into prenatal care. In addition, sexual IPV was associated with late entry into prenatal care, urinary tract infections, and low birth weight. Moreover, IPV during pregnancy/postpartum might increase the risk of developing severe mental illnesses in both victims and sons (23).

Moreover, several women victims of violence may experience a re-exacerbation of pre-existent psychiatric disorders, with a considerable risk of disorder' chronicity and increased severity (20). It has been demonstrated that women with severe psychiatric disorders are at increased risk of becoming domestic violence victims: this may be particularly true during the pandemic (24). A large Swedish registry study found that, compared to general population controls, all psychiatric diagnoses studied (except autism) were associated with an increased risk of domestic violence against women in men (25). Therefore, particular attention should be given by psychiatrists to such persons during the pandemic.

Moreover, a woman who is a victim of violence may quickly develop suicidal ideation that may elicit suicidal behaviors, especially when the violence is frequent and she has no way to escape or get help and support (26). Therefore, the existence of clinically manifest suicide ideation, independent of current psychopathology, must always be actively evaluated and adequately addressed (27).

All these issues may also take into account the psychological distress generated by the COVID-19 (i.e., fear of contagion and death, worrying about close relatives, sleep disturbances, forced inactivity, binge eating, etc.), triggering a vicious circle wherein violence' consequences overlap on COVID-19 psychological anguish, thus potentially enhancing each other. Moreover, the children of maltreated women may be at risk of developing psychiatric consequences that may impact their lives and their future, as they may also be victims of violence by the same perpetrator or even witness acts of violence on beloved ones (28).

It is worthy of note that, in some countries, the social and economic disparities might further impact women, thus increasing the possibility of being a victim of violence in such less disadvantaged contexts (29).

## Violence Against the Women During COVID-19 Pandemic: A Call of Action

To date, psychiatrists and mental health workers should be organized and prepared to evaluate this new potential gender-based “psychiatric” pandemic. Health and mental health facilities should systematically search for potential warning signs of VAW, improving recognition, management, and referral pathways for sufferers. Such signs might include partner bullies, threatens, controls, partner' cutting off women from family and friends, strict control on women's money and financial incomes, objective signs of physical abuse and beating, etc. In addition, we believe that all health workers need to be appropriately trained in diagnosing trauma-related conditions and conducting a dialogue with the victim to detect them (13).

The psychiatric services must work together with the anti-violence centers, which should be rapidly empowered with trained personnel and financial support. Moreover, telehealth should be implemented (30) as it is a suitable instrument to give easy-to-use and affordable support through several web platforms that have demonstrated usefulness during the lockdown (31). Several studies have demonstrated that telehealth interventions might help detect women's health concerns, including violence and IPV (32–34). Psychological approaches to depressive and post-traumatic symptoms through supportive psychotherapy, cognitive behavior therapy, and interpersonal therapy are particularly significant and should be provided to all victims.

Moreover, substance use disorders, as primary or comorbid diagnoses, are associated with the highest absolute and relative risks of domestic violence perpetrated by men (35), so treatment for these, together with any comorbid psychiatric disorder, should be prioritized working together with the centers for the Addictions (36).

However, we firmly believe that cultural change and prevention campaigns are urgently needed. Bellizzi et al. (9) stated there is a need for several countries to guarantee that policies and measures equally address prevention, protection, investigation, and punishment that require coordination between national, regional, and local authorities. The “RESPECT women” document of WHO seems a useful model on which to build effective strategies (R–elationship skills strengthened; E–mpowerment of women; S–ervices ensured; P–overty reduced; E–nvironments made safe; C–hild and adolescent abuse prevented; T–ransformed attitudes, beliefs, and norms) (37). However, in some cases, specific barriers for women to access services and for health care utilization exist, including minimization of forms of abuse, perceptions of abuse and violence as normal, lack of awareness of services, the fear for the lack of confidentiality and stigma and the poor social and community support (38) and these should be overcome with specific campaigns and targeted interventions.

Finally, worldwide communities must be aware and conscious of this phenomenon, improving knowledge and advocacy through such national campaigns and projects, as often neighbors and friends may be the first line of communication for sufferers during lockdown or restrictions. These preventive strategies to contrast the violence against women help give details on help requests and management pathways.

Moreover, greater attention should be given to contexts in which this phenomenon is widespread, even not adequately studied, as among female migrant populations (39). In such populations, due to the multitude of risk factors that women living in these contexts already face up to, COVID-19 negative impact and consequences on their mental health, including violence against them, could be even worse, and every effort should be made to prevent it.

## Author Contributions

All authors have contributed to this paper with equal efforts.

## Conflict of Interest

The authors declare that the research was conducted in the absence of any commercial or financial relationships that could be construed as a potential conflict of interest.
